# Manitoba Veterinary Association

**Published:** 1897-04

**Authors:** 


Secretary.

### MANITOBA VETERINARY ASSOCIATION.

THE Provincial Veterinarians met at Winnipeg, along with other associations. President M. Young, of Manitou, in the chair. For the ensuing year the Directors chosen were: Drs. Young, Torrance, Dunbar, Rutherford, Thompson, Swinnerton, and Hinman. The Directors elected the following officers: President, W. J. Hinman; Vice-President, F. Torrance; Secretary-Treasurer and Registrar, W. A. Dunbar; Examining Officers, Messrs. Hinman, Torrance, and Dunbar; Auditors, Drs. Hinman and Thompson. Several interesting professional papers were read and discussed. A committee composed of Drs. Torrance, Young, and Rutherford was appointed to wait on the Pure-bred Cattle Breeders' Association and urge that Association to take action in the matter of tuberculosis inspection and certificates. It was decided that the next meeting of the Veterinary Association should take place at Winnipeg during the time of the Provincial Exhibition.

